# Investigation of the Quality of the 12 Most-Used Antibiotics Available in Retail Private Pharmacies in Rwanda

**DOI:** 10.3390/antibiotics11030329

**Published:** 2022-03-02

**Authors:** Thomas Bizimana, Védaste Kagisha, Jean Baptiste Nyandwi, Alain Katembezi Nyirimigabo, Raymond Muganga, Marie Françoise Mukanyangezi, Egide Kayitare

**Affiliations:** 1 Department of Pharmacy, School of Medicine and Pharmacy, College of Medicine and Health Sciences, University of Rwanda, Kigali P.O. Box 3286, Rwanda; kagishaveda@gmail.com (V.K.); nbaptiste1988@gmail.com (J.B.N.); mugangaray@gmail.com (R.M.); francizi@yahoo.fr (M.F.M.); egide.kayitare@gmail.com (E.K.); 2 Division of Quality Control Laboratory, Rwanda Food and Drugs Authority, Kigali P.O. Box 1948, Rwanda; akatembezi@rwandafda.gov.rw

**Keywords:** quality control, antibiotic, visual inspection, counterfeit, falsified medicines

## Abstract

Using poor-quality antibiotics leads to increased risk of the development of microorganism-resistant strains, treatment failure, loss of confidence in health systems, and associated socio-economic impacts. The prevalence of poor-quality antibiotics has been found to be high in some of the Low and Middle-Income Countries (LMICs), but no data were available on the situation in Rwanda. This study was conducted to obtain data and inform health professionals on the quality of the 12 most-used selected antibiotics from private retail pharmacies in Rwanda. The investigation was conducted on 232 batches collected from randomly selected private retail pharmacies in all provinces of Rwanda, and concerned only with visual inspection and assay tests. Visual inspection was performed using a tool adopted by the International Pharmaceutical Federation (FIP) to identify manufacturing defects. An assay test quantified the Active Pharmaceutical Ingredient (API) in each collected batch using high-performance liquid chromatography (HPLC) coupled with an ultraviolet-visible (UV) detector, and the results were reported as the percentage content of the amount of APIs stated on the label. A total of 232 batches were analyzed, manufactured in 10 countries; the main country of manufacture was Kenya, with almost half of the batches (49.6%). The results of the visual inspection did not show the presence of counterfeit/ falsified antibiotics on the Rwandan market in this study but revealed weaknesses in labeling: more than 90% of the analyzed batches of the 12 antibiotics did not present the dosage statement on their label, and the complete list of excipients was missing in more than 20% of the analyzed batches. The assay test using HPLC confirmed the presence of APIs in 100% of the analyzed batches. However, moderate deviations from acceptable ranges of the API content defined by M. M. Nasr & C. M. Stanley in 2006 for erythromycin and the United States Pharmacopoeia 2018 for the other 11 molecules were found. The failure rate to meet the quality requirements in terms of the percentage content of active pharmaceutical ingredients declared on the labels was estimated at 8.2% in total, with 3.9% and 4.3% containing more and less than the amount of APIs stated on the labels respectively. The most-represented antibiotics on the Rwandan market were amoxicillin, co-trimoxazole and cloxacillin. No counterfeit antibiotics were found in this study. However, substandard batches with moderate deviations were found, suggesting that regular quality control of antibiotics is needed in Rwanda.

## 1. Introduction

Since the 1980s, there have been very few new antibiotics [1]. If bacteria continue to develop resistance to existing antibiotics, there is a risk of losing stronger ones to treat serious infections. Resistant bacteria are spread and shared all over the world, and can affect anyone, of any age, in any country [2]. Factors including the use of substandard antibiotics and irrational use of antibiotics influence the development of resistant bacteria [3], and this has become a global health and development threat, hindering the achievement of Sustainable Development Goal 3.1 (SDG3.1) defined by the United Nations (UN) by 2030 [4].

The presence of substandard and counterfeit antibiotics on the global market constitutes a big concern to public health and socio-economic development [5]. The risks associated with the use of substandard antibiotics affect health, economy and socioeconomic aspects [6,7,8]. Not only do substandard antibiotics fail to treat an infection, the patients also risk experiencing toxicity, serious side effects, the development of strains resistant to the antibiotic used, loss of productivity, unnecessary expenses to treat side effects, complication of the infection that can lead to death, and loss of confidence in the health system [7,9].

The actual trend in the prevalence of substandard antibiotics is alarming and globally united efforts are required [10,11]. The most targeted classes of medicines in LMICs are antibiotics, anti-tuberculosis medicines, antimalarials, and antiretrovirals [12,13,14]. A study, published in 2020 by Simon Schäfermann et al., reported an overall failure rate of 25.3% with reference to the United States Pharmacopoeia (USP) for samples of antibiotics collected from government and faith-based health facilities, private pharmacies, and informal vendors (total 60 facilities) of Cameroon and the Democratic Republic of Congo (DRC), analyzed using HPLC [15]. Similarly, in a recent study conducted in Haïti by Théodule Jean-Baptiste et al. on 11 antibiotics using a Raman handheld spectrometer, none of the tablets/capsules for six antibiotics (Tetracycline, Erythromycin, Cloxacillin, Azithromycin, Clarithromycin, and the combination Amoxicillin + Clavulanic Acid) showed a sufficient spectral match with the authentic medicine [16]. With reference to the USP, Eva Hobeika et al. analyzed samples of ciprofloxacin, amoxicillin and amoxicillin-clavulanic acid collected from the Lebanese market in 2020 using HPLC for the assay limit of the Active Pharmaceutical Ingredient (API). All five samples of ciprofloxacin failed, and therefore, were substandard [17]. Samuel Oppong Bekoe et al., in a study conducted in Ghana in 2020 on 348 samples of 14 antibiotic molecules, found that the failure rate of antibiotics was 66.4% [18].

The literature review has revealed the unavailability of results showing the quality of antibiotics used in Rwanda. As counterfeit and substandard antibiotics are likely to be found in LMICs, the research team from the Department of Pharmacy at the University of Rwanda designed this study aiming at filling in missing data and informing stakeholders in the fight against counterfeit/falsified and substandard antibiotics. Specifically, the study was to visually inspect a maximum number of batches of antibiotics available in Rwanda and to evaluate the quality of 12 selected antibiotics available in the private retail pharmacies of Rwanda with the aim to assess the exposure of patients to poor-quality antibiotics and proposing appropriate actions.

## 2. Results

### 2.1. Overview of Analyzed Antibiotics

In total, 12 antibiotics comprising 232 different batches distributed under 72 different brand names and 18 batches without brand names were assessed. The findings of this study show that about half (49.6%) of the analyzed batches were manufactured in Kenya, 20.7% were manufactured in India and 12.5% were manufactured in China. Few batches of the analyzed antibiotics were manufactured in European countries known as Stringent Regulatory Authorities (SRAs), namely France (8.6%), Turkey (2.2%), Germany (2.2%) and Italy (0.4%). The other countries of origin were Uganda (2.6%), Morocco (0.9%) and Senegal (0.4%).

The 232 batches were distributed as follows: 84 were tablets, 58 were capsules, 50 were powders for oral suspension, and 23 were oral suspensions, whereas powders for injection were represented by 14 batches and only three batches of solutions were included (Table S1). The distribution of batches by molecules was as follows: amoxicillin (26.7%), Co-trimoxazole (15.9%), Cloxacillin (13.4%), Erythromycin (10.8%), Ciprofloxacin (8.6%), Doxycycline (8.2%), Metronidazole (7.3%), Phenoxymethylpenicillin (3%), Ampicillin (2.2%), Ceftriaxone (1.7%), Cefotaxime (1.3%) and Amoxicillin + Clavulanic acid (0.9%).

### 2.2. Compliance with Visual Inspection Parameters of 12 Antibiotics

A counterfeit medicine is “deliberately and fraudulently mislabeled with respect to identity and/or source. Counterfeiting may include products with the correct ingredients or with the wrong ingredients, without active ingredients, with insufficient active ingredients or with fake packaging” [19]. The compliance to visual inspection parameters was expressed in percentages for each assessed parameter and the number of asked questions (Table S2).

#### 2.2.1. Summary Percent Compliance of Pharmaceutical Dosage Forms to Visual Inspection Parameters

**Figure 1:**
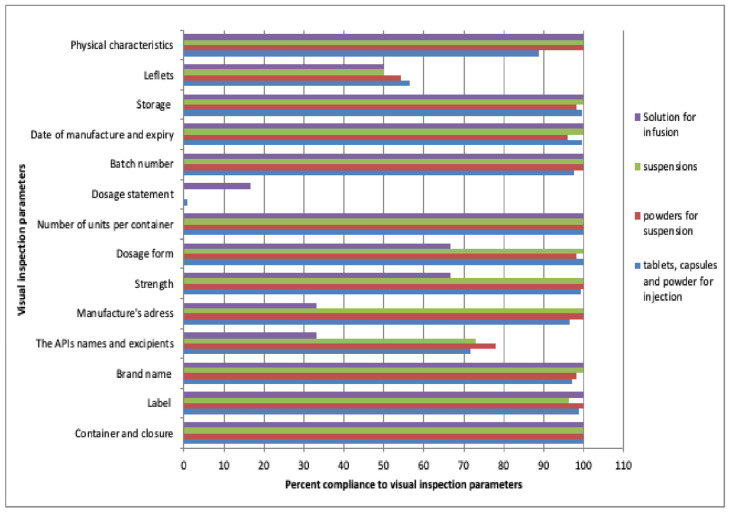
Compliance with visual inspection parameters for all considered pharmaceutical dosage forms: tablets, capsules, powder for injection, powders for oral liquid formulation, oral liquid formulations and infusion.

The results of the visual inspection (Figure 1) showed that, in this study, none of the pharmaceutical dosage forms fully complied with all visual inspection parameters at 100%.

Most of the parameters (10/14) complied at a percentage above 95% for all of the pharmaceutical dosage forms. More than 99% of the stated manufacturers failed to mention the dosage statement, except for metronidazole infusion, and incomplete information on leaflets was found in more than 40% of batches. Moreover, a complete list of APIs and excipients was observed on 78% of powder for oral liquid formulation, 73.1% for oral liquid formulation and 71.4% for tablets, capsules and powder for injections.

#### 2.2.2. Percentage Compliance of Antibiotics to Visual Inspection Parameters

The percentage compliance of antibiotics with visual inspection parameters was determined by calculating the overall coverage of 14 parameters (Table S2). None of the analyzed batches complied totally with visual inspection parameters. However, most of the batches (168/232) scored a percentage compliance between 70% and 90% and few (64/232) exceeded 90% (Figure S1).

In addition, during the preparation phase of the HPLC samples, it was observed that, for some brands of amoxicillin and cloxacillin capsules, the powder had solidified to the extent where the formed solid was not removed from the opened capsule by gravity unless it was broken into pieces (Figure S2). Results of the disintegration and dissolution tests would have provided additional information on the quality of these samples. Unfortunately, these two tests were not part of the scope of this study, and therefore, were not performed.

### 2.3. Percent Content in Active Pharmaceutical Ingredients of Analyzed Antibiotics

Substandard medicines are defined as “genuine medicines produced by legitimate manufacturers that do not meet the quality specifications that the producer says they meet. For example, they may contain less (or more) active ingredients than written on the package. This may not be an intention to cheat, but may be due to problems with the manufacturing process” [19]. “Degraded medicines may result from exposure of good-quality medicines to light, heat, and humidity. It can be difficult to distinguish degraded medicines from those that left the factory as substandard, but the distinction is important as the causes and remedies are different” [19].

The results of HPLC analysis are presented by individual batches analyzed with reference to the United States Pharmacopoeia (USP) [20] acceptance ranges for 11 antibiotics, and erythromycin was analyzed with reference to the method developed and published by M. M. Nasr & C. M. Stanley in 2006 [21]. Acceptance ranges for the content in active pharmaceutical ingredients were 93–107% for co-trimoxazole tablets; 90–110% for ciprofloxacin tablets, co-trimoxazole oral suspension, metronidazole tablets, metronidazole oral suspension and metronidazole intravenous solution; 90–115% for ampicillin powder for injection, cefotaxime powder for injection, and ceftriaxone powder for injection; 90–120% for amoxicillin capsule, amoxicillin powder for oral suspension, amoxicillin + clavulanic acid tablets, cloxacillin capsule, cloxacillin powder for oral suspension, cloxacillin powder for injection, doxycycline tablets, doxycycline capsule, erythromycin tablets, erythromycin powder for oral suspension and phenoxymethylpenicillin tablets [20].

**Figure 2:**
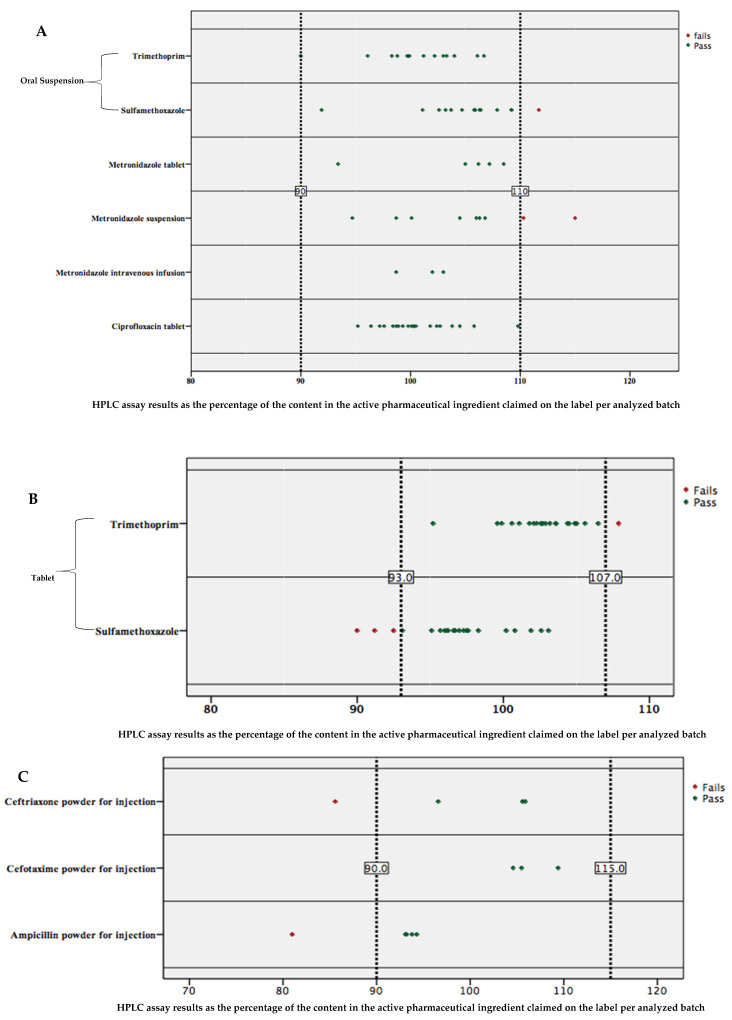

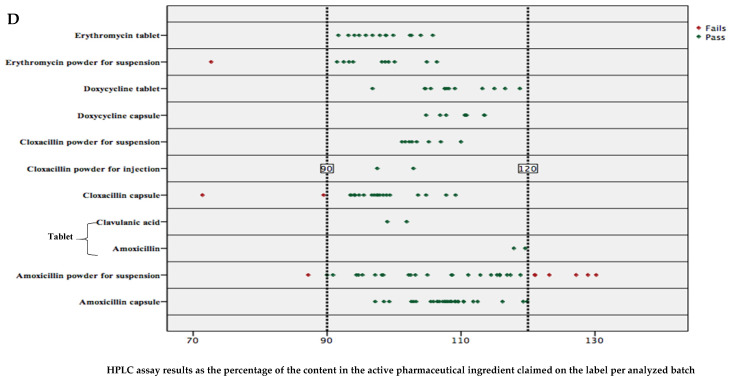
Percentage API content by batches according to different acceptance ranges as defined by the USP for each analyzed antibiotic, (**A**) 90–110, (**B**) 93–107, (**C**) 90–115 and (**D**) 90–120. Each point represents a batch, but one point (batch) of amoxicillin powder for oral suspension is hidden by another batch which scored a close result for the same antibiotic (121.0% and 121.1%).

The results of the HPLC analysis (Table S1) show the failure rate by molecules as follows: Co-trimoxazole 5/37 (13.5%), Metronidazole 2/17 (11.8%), and amoxicillin 7/62 (11.3%), as the failure rate within the total number of batches for an individual antibiotic. No batches of Ciprofloxacin, Phenoxymethylpenicillin, Amoxicillin + Clavulanic acid, Doxycycline, and Cefotaxime failed to meet pharmacopeial requirements for the assay content test. The overall failure rate of antibiotics was found to be 8.2%, with moderate deviations above the upper limit (4.3%) and below the lower limit (3.9%) (Figure 2), therefore they were substandard. This study did not cover impurity of degradation; consequently, no degraded antibiotics were reported.

## 3. Discussion

As previously reported in different studies carried out in other LMICs [22,23,24,25], the results obtained from the visual inspection did not reveal any counterfeit/ falsified medicines. Our study failed to obtain genuine samples from manufacturers to compare to our samples, as was also the case in a study conducted in Ghana, Nigeria and the United Kingdom by Fadeyi Ifeyinwa et al. in 2015 [26]. Therefore, the findings from this study do not give a total guarantee of a market free from counterfeit antibiotics, since in some LMICs, counterfeit medicines have been found [15,27,28,29]. The main information from the visual inspection, namely: the quality of containers and closure systems, the indication on the number of units per container, the presence of labels, indication of the brand names, batch number, expiry dates, and storage conditions complied with a good score (97–100%) for all collected dosage forms, differently from what was found in the Lao People’s Democratic Republic in 2019 [10]. This study shows that some important information has a very low score, between 10 and 33%. This is the case for the dosage statement, indication of the used excipients, and the manufacturers address. The list of excipients should be written on the label [30]. Furthermore, the solidification of powder observed in the capsules was probably due to either poor manufacturing practices or poor storage conditions. Poor storage conditions of amoxicillin and cloxacillin may result in solidification of the powder and lead to bacterial resistance [31]. Interestingly, the same batches also showed a low percentage content of APIs, suggesting that their concentrations may have been reduced.

The active pharmaceutical ingredients were present in all the samples tested. However, there were two types of moderate deviations: batches with lower and batches with higher than the acceptable content of APIs. The antibiotics with higher API contents were the most observed. Fortunately, the rate of substandard antibiotics found in this study was less than the one reported in a previous study carried out in Ghana [18] and also less than the average of 25% according to the review published in 2020 by Dominic McManus and Bernard David Naughton [32]. Similarly to the study performed in the Lao People’s Democratic Republic in 2019 [10], all except one batch contained between 75% and 125% APIs, proving that there were no extreme deviations from the content stated on the label. However, this does not provide enough evidence to protect the Rwandan population against antimicrobial resistance and the risks of toxicity, since suboptimal doses and high blood concentrations are likely to be experienced. In this study, 36.8% of substandard antibiotics were amoxicillin, which was also the most-represented antibiotic (26.7%) and its overall failure rate was estimated at 11.3%. A lower percentage failure rate was found in Ghana and Nigeria in 2015 [26], while a percentage failure rate higher than the one found in our study was observed in Papua New Guinea in 2021 [25] and the Lao People’s Democratic Republic [10]. The percentage failure for doxycycline in this study was different from the situation in the Democratic Republic [15,22]. Substandard ciprofloxacin medicines were absent in this study, differently from the results found in Myanmar in 2020, where the failure rate of ciprofloxacin was 16% [29]. The quality of batches of sulfamethoxazole + trimethoprim was found to be improved in Rwanda compared to other LMICs. Ifeyinwa Fadeyi et al. found that 60% of co-trimoxazole tablets purchased in Ghana and Nigeria in 2015 did not meet quality requirements [26]. Other authors found substandard antibiotics in different LMICs [25].

The Rwandan market is not safe from the entrance of substandard antibiotics. Even though no counterfeit antibiotics were declared on the Rwandan market, the supply chain for antibiotics needs regular monitoring and timely quality control to ensure improved accessibility for users in Rwanda to good-quality antibiotics. Visual inspection has to be performed whenever a health professional is in contact with medicines, to detect the presence of counterfeit/falsified antibiotics early. In addition, the competent authority should avail genuine samples for brands of antibiotics authorized in Rwanda, to serve as reference samples during a comprehensive investigation on antibiotics.

All required pharmacopeial tests were not performed; the present study focused on the visual inspection and the assay tests.

## 4. Materials and Methods

### 4.1. Study Design

This study was cross sectional, quantitative and descriptive, targeting 12 antibiotics commonly used in Rwanda, selected and collected from Kigali city and all provinces of Rwanda. The list of commonly used antibiotics investigated in this study was confirmed in collaboration with the Ministry of Health (MoH), Rwanda. Samples were collected in 2018 and concerned the following medicines: amoxicillin, amoxicillin/clavulanic acid, erythromycin, ceftriaxone, cloxacillin, ampicillin, cefotaxime, phenoxymethylpenicillin, ciprofloxacin, sulfamethoxazole/trimethoprim, metronidazole and doxycycline. Visual inspection using an adapted tool from FIP and assay/content, using HPLC methods, was performed. The guidelines of MEDQUARG and the WHO on the conduct of surveys on the quality of medicines were observed during the design and implementation of this study.

### 4.2. Samples Collection

Samples were purchased in 2018 from 84 private pharmacies open to the community, located in 18 districts randomly selected from the list of 30 districts of Rwanda using the RAND function of Microsoft Excel 2018. In addition, the three districts of the capital city of Rwanda, Kigali were also included. In each of the 18 districts, one retail pharmacy was selected in the center of the district city, then four more retail pharmacies were selected in four directions from the center of the district city. In the capital city of Rwanda, where more than 60% of retail pharmacies are concentrated, 15 retail pharmacies were selected randomly in each of the three districts.

At each pharmacy, the following criteria were considered for a sample to be collected: for different pharmaceutical dosage forms, different strengths and different brands of each of the 12 selected antibiotics, a batch was collected once if the required quantity was available. Different sample collectors had to check the online shared google sheet, in which they were entering descriptions of the samples and data for visual inspection, to find out whether the new batch had been collected yet or not. The number of collected units by sample depended on the pharmaceutical dosage form: 100 tablets or capsules, 10 ampoules/vials and 5 bottles of oral suspensions, powder for oral suspension, solutions or powder for injection.

### 4.3. Visual Inspection

The visual inspection assessment concerned 14 parameters: container and closure, label, brand name, active ingredient name and excipients, manufacturer’s full address, strength, dosage form, number of units per container, dosage statement, batch number, date of manufacture and expiry, storage, leaflet, physical characteristics (Table S2). Data for visual inspection were collected during the process of sample collection in 2018, and re-checked at the time of the assay test in 2019–2020.

In Rwanda, the National Regulatory Authority (NRA) is not in place. Responsibilities of the NRA are currently fulfilled by the Rwanda Food and Drug Authority (RFDA), which was a new institution in 2018 when this study started, established by the law Nº 003/2018 of 09/02/2018 [33]. The RFDA started functioning in 2018, and only a few medicines have gone through the complete process of registration with genuine samples submitted and stored [34]. All brands of medicines used in Rwanda before RFDA started its activities were not completely registered, and they are still marked as authorized in Rwanda [35]. Therefore, it was not possible to find genuine samples from manufacturers of the different brands of the 12 antibiotics collected in Rwanda. The research team adapted the “Tool for visual inspection of medicines” developed by the “International Council of Nurses in partnership with the United States Pharmacopoeia (USP)” and modified by the “Military and Emergency Pharmacists Section of FIP” to fit the situation.

### 4.4. HPLC Analysis

Assay tests were performed in 2019 and in 2020 for all batches analyzed for visual inspection. HPLC analytical methods specific to each of the 12 antibiotics from the United States Pharmacopoeia 2018 were adopted and used.

#### 4.4.1. Instrumentation and Chemicals

Samples were analyzed in the Huye Biotechnology Laboratory Complex, Rwanda, using the Agilent Technologies 1200 series HPLC system. The HPLC system was equipped with a vacuum degasser, Quaternary Pump, Column Oven, Autosampler and Diode Array Detector. Data were analyzed using the Chemstation Manager software. A 1100L pH meter was used for pH measurements and adjustments. Other small equipment used include an ultrasonic bath, centrifuge machine and Quintix 124-1S Analytical balance. The reference standards of amoxicillin trihydrate, ceftriaxone sodium, cefotaxime, doxycycline, ampicillin, erythromycin, clavulanic acid, cloxacillin sodium, metronidazole, phenoxymethylpenicillin potassium salt, ciprofloxacin, sulfamethoxazole, and trimethoprim were purchased from Sigma Aldrich, Germany. Water of HPLC grade was produced in Huye Biotechnology Laboratory Complex, Rwanda. Methanol and acetonitrile of HPLC grade were purchased from Merck, Belgium. Potassium Dihydrogen Phosphate of AR grade was purchased from Sigma Aldrich, Germany. The aqueous phase was filtered through a 0.45 µm membrane filter and sonicated before use.

#### 4.4.2. Samples Preparation and Analysis

During the samples’ preparation phases, both samples and standards were prepared targeting a final concentration of 100 µg/mL, excepting combinations. For tablets and capsules, the samples were prepared by dissolving an appropriate mass of powder weighed from the powder obtained by emptying 10 capsules or grinding 10 tablets. Amoxycillin, ampicillin, amoxicillin/clavulanic acid, cefotaxime, ceftriaxone, were dissolved in water, ciprofloxacin was prepared in the mobile phase of the method used for its analysis, cloxacillin was prepared in a mixture of KH_2_PO_4_ 0.01M, pH = 6.8/MeCN (70/30), erythromycin samples were prepared in ACN: H_2_O mixture (50:50), and sulfamethoxazole/trimethoprim was prepared in a mixture of MeOH: H_2_O (50:50) (Table S3). In all cases, sample solutions were sonicated for 15 min to ensure complete dissolution and then filtered through a 0.45 μm filter.

For oral suspensions and injectables, the injected solutions were prepared from the reconstituted oral suspensions as indicated by the manufacturer. Before pipetting from the reconstituted oral suspension, it was shaken vigorously manually to insure the homogeneity of the sample. The pipetted samples were diluted using the appropriate solvent as previously mentioned. The obtained solution was filtered with a 0.45 μm membrane filter before injection in HPLC and were analyzed and repeated three times. After the analysis, the API contents were calculated based on the areas under the peaks and by applying the single-standard method. The obtained results were reported as percentages and were compared with the acceptance limit for each product stated in the USP 2018.

### 4.5. Data Analysis

Microsoft Excel software was used for data curation and descriptive statistics. The Statistical Package for the Social Sciences (SPSS) was used for statistical analysis.

## 5. Conclusions

Amoxicillin, co-trimoxazole and cloxacillin were the most-represented in the collected batches. No counterfeit medicines were identified on the Rwandan market during this study. The results from HPLC analysis allowed us to check if the analyzed medicines contained an acceptable level of active ingredients. Although the majority of analyzed medicines meet the requirements, some batches are outside of the acceptable ranges and moderate deviations from the optimal percent content of the declared amount of active pharmaceutical ingredients were found. This is the case for some batches of tablets and oral suspension of co-trimoxazole, metronidazole oral suspensions, ampicillin for injection, ceftriaxone for injection, amoxicillin oral suspension and capsules of cloxacillin. Some batches of co-trimoxazole tablets and capsules of cloxacillin contained an insufficient level of the active pharmaceutical ingredient. On the other side, most of the substandard batches exceeding the acceptable level of active pharmaceutical ingredients were found in powders for oral suspension of amoxicillin, metronidazole and co-trimoxazole. As the treatment of infections using substandard antibiotics has a negative impact on the health of the population and the economy, systematic and regular quality control of antibiotics entering the Rwandan market is highly recommended.

## Supplementary Materials

The following are available online at https://www.mdpi.com/article/10.3390/antibiotics11030329/s1, Figure S1: Brand names and the percent compliance to visual inspection for (A) Tablets; (B) Intravenous infusion, powder for suspension and suspensions and (C) Capsules and powder for injection; Figure S2: Pictures of solidified powder of (A) Amoxicillin and (B) cloxacillin; Figure S3: Sample chromatograms for (A) Cloxacillin; (B) Phenoxymethylpenicillin; (C) Ciprofloxacin; (D) Cotrimoxazole; (E) Amoxicillin; (F) Ceftriaxone; (G) Doxycycline; (H) Metronidazole; (I) Ampicillin; (J) Amoxicillin + clavulanic acid; (K) Erythromycin; Table S1: Summary of analyzed batches and HPLC results; Table S2: Adapted tool for visual inspection; Table S3: HPLC chromatographic conditions.
