# Supplementary material for: Investigation of the Quality of the 12 Most-Used Antibiotics Available in Retail Private Pharmacies in Rwanda
Source: Antibiotics (Basel). 2022 Mar 2;11(3):329. doi: 10.3390/antibiotics11030329 (PMC8944805; doi:10.3390/antibiotics11030329)
Supplement: Supplementary file 1 [file antibiotics-11-00329-s001.zip › antibiotics-1548775-supplementary.pdf]

Supplementary files

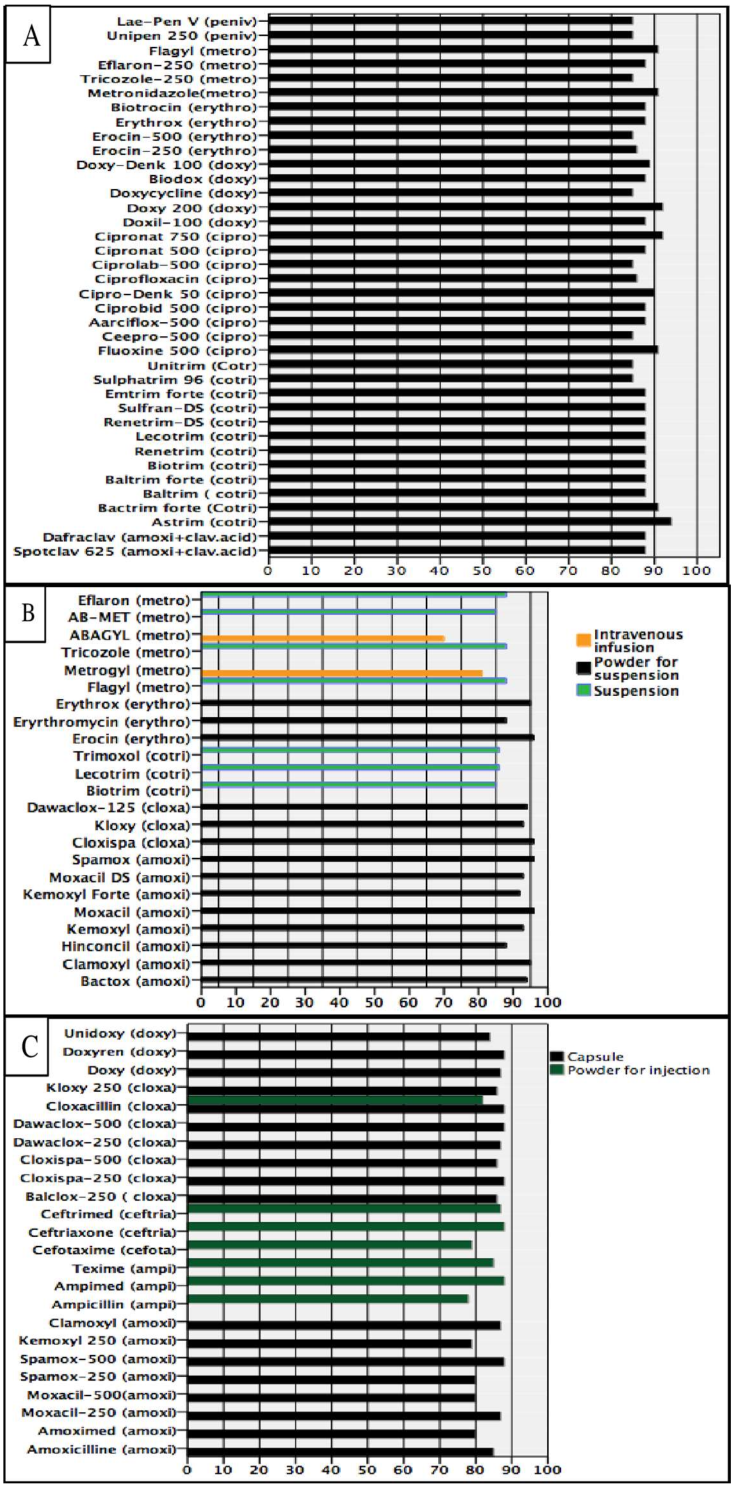

Figure S1. Brand names and the percentage compliance to visual inspection for (**A**) tablets; (**B**) intravenous infusion, powder for suspension and suspensions and (**C**) Capsules and powder for injection.

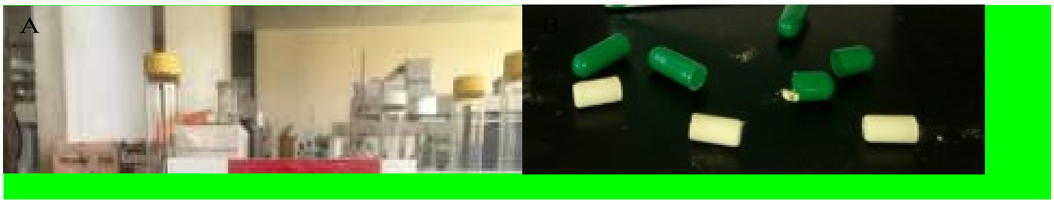

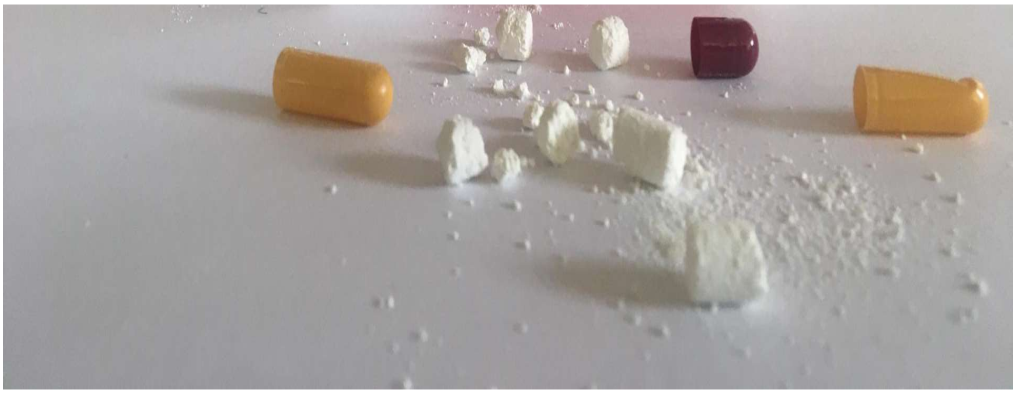

Figure S2A. Picture of solidified powder of Amoxicillin

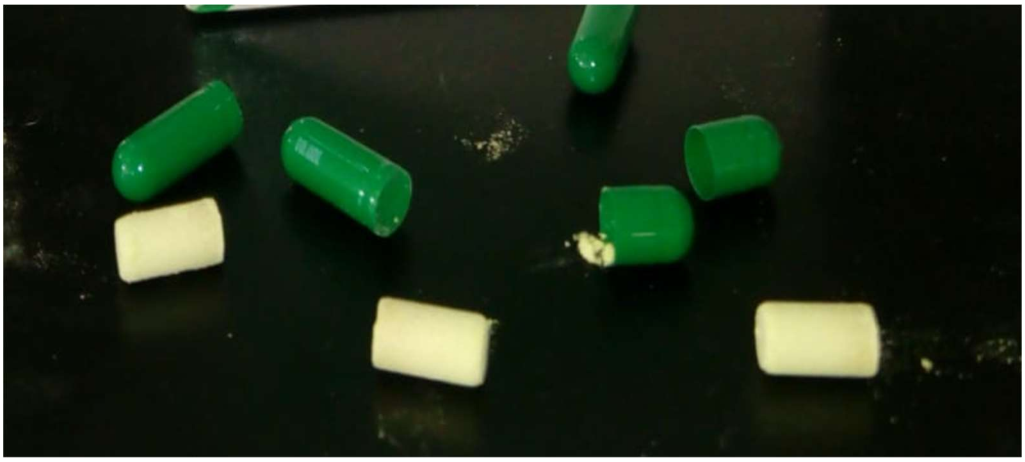

Figure S2B. Picture of solidified powder of cloxacillin.

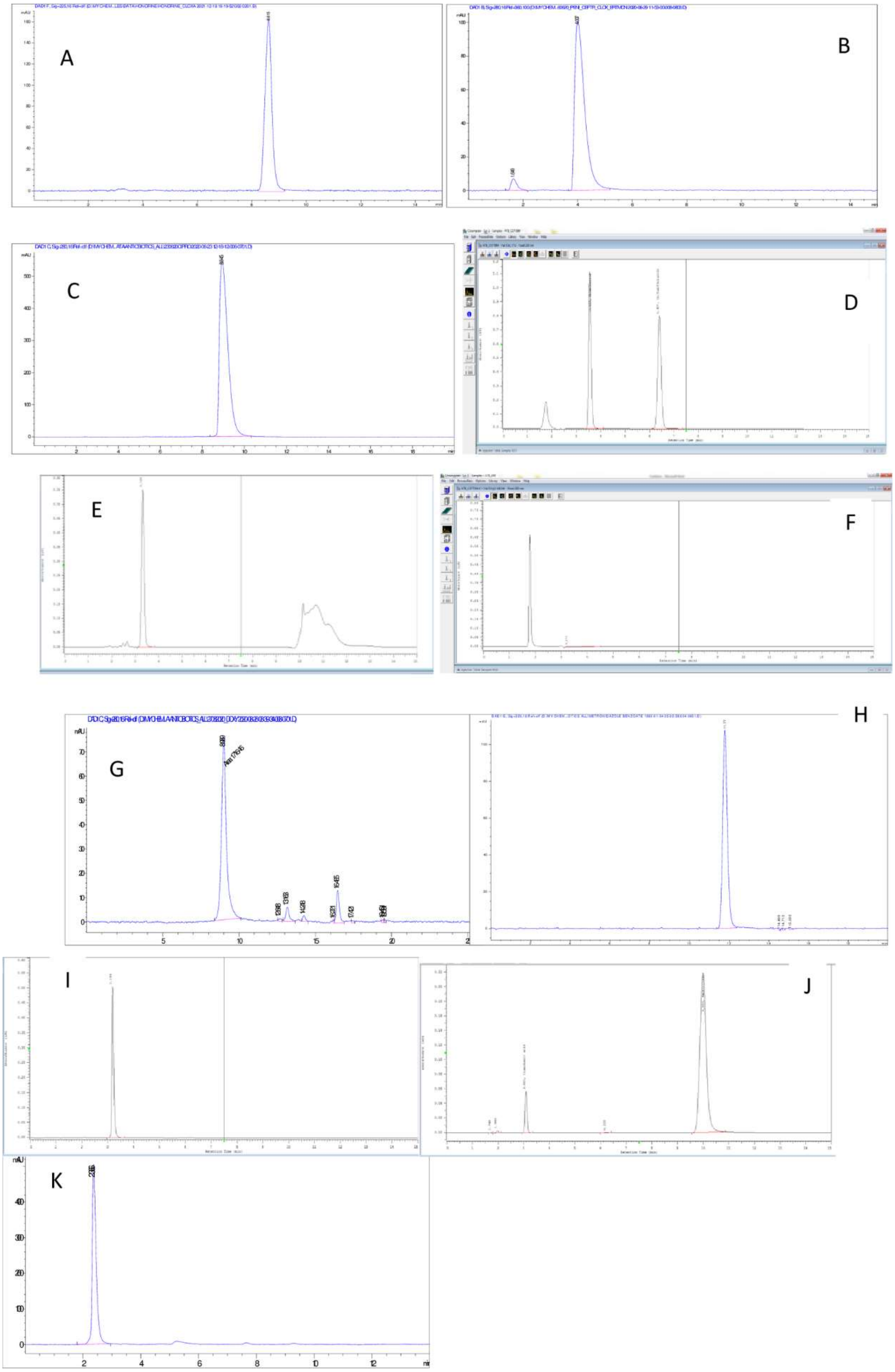
A
B
C
D
E
F
G
H
I
J
K

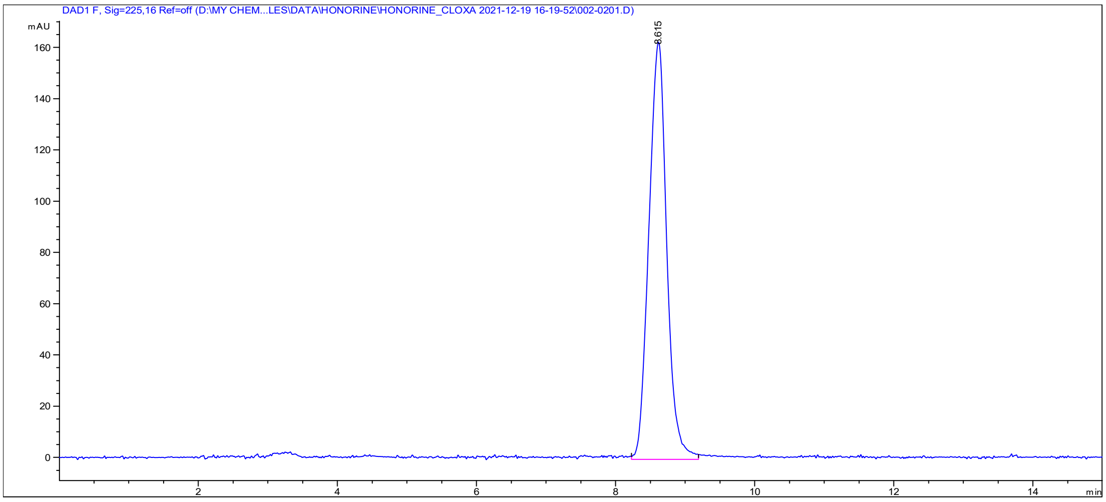

Figure S3A. Sample chromatogram for Cloxacillin

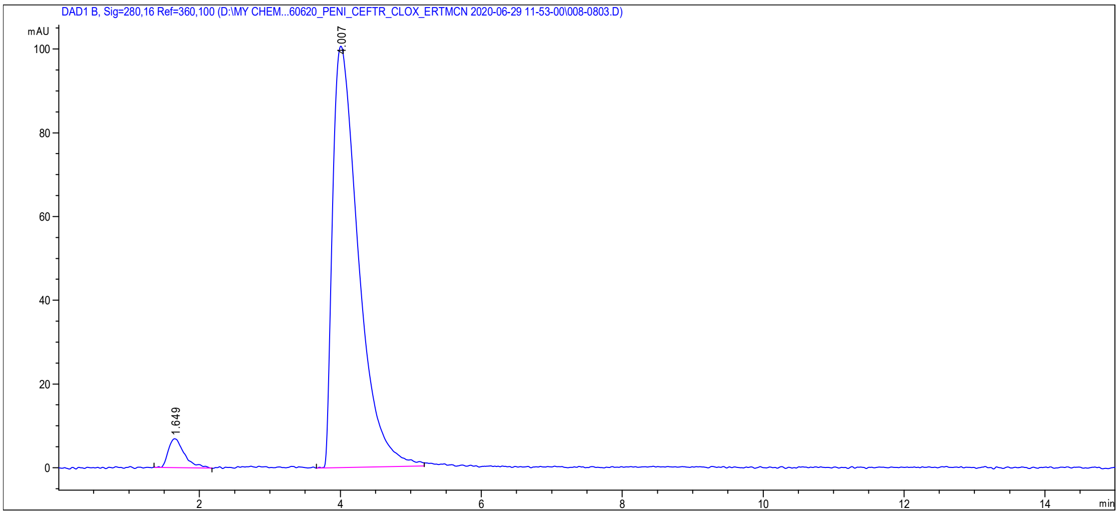

Figure S3B. Sample chromatogram for Phenoxymethylpenicillin

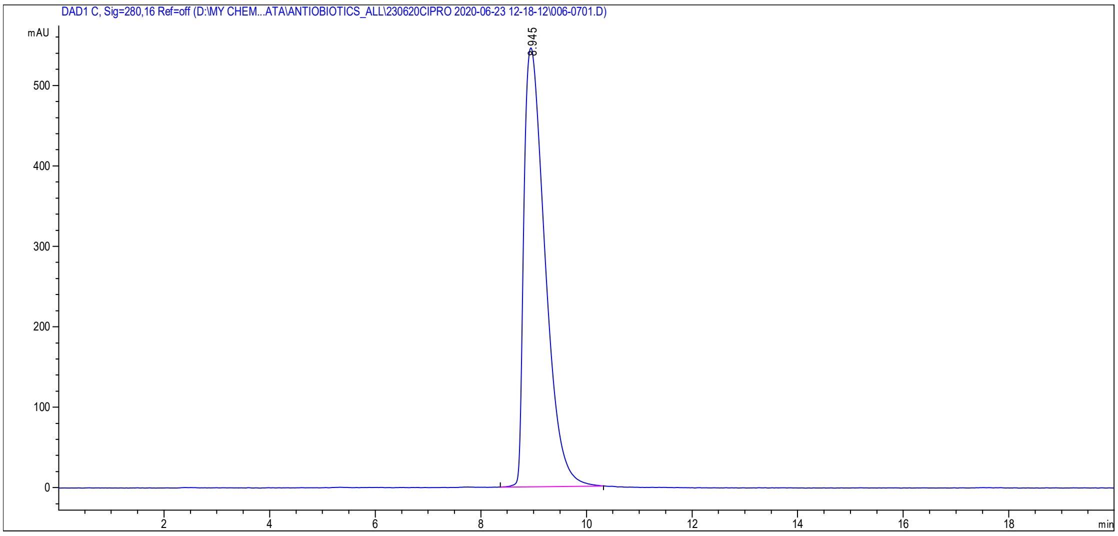

Figure S3C. Sample chromatogram for Ciprofloxacin

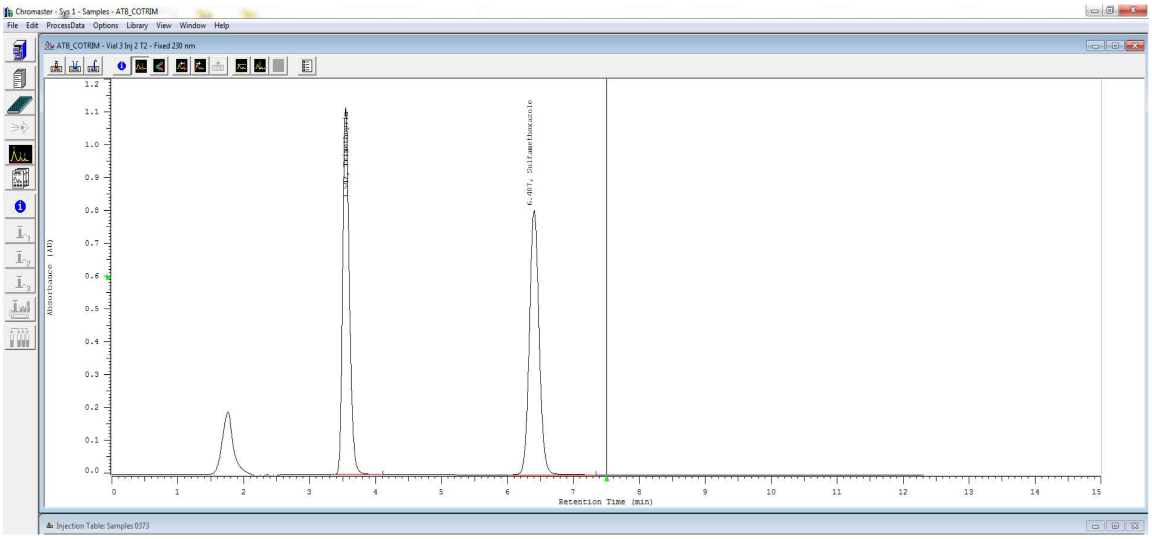

Figure S3D. Sample chromatogram for Cotrimoxazole

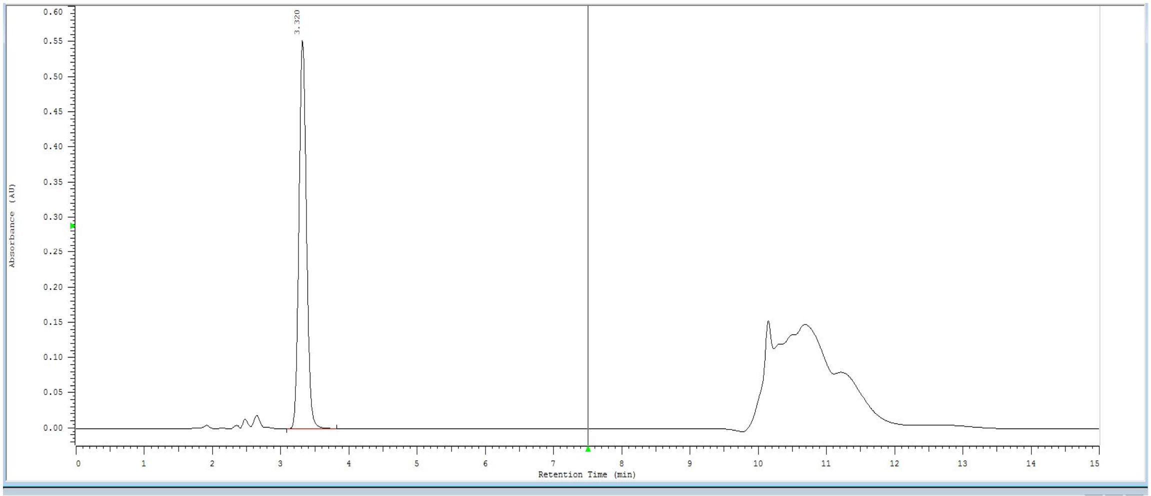

Figure S3E. Sample chromatogram for Amoxicillin

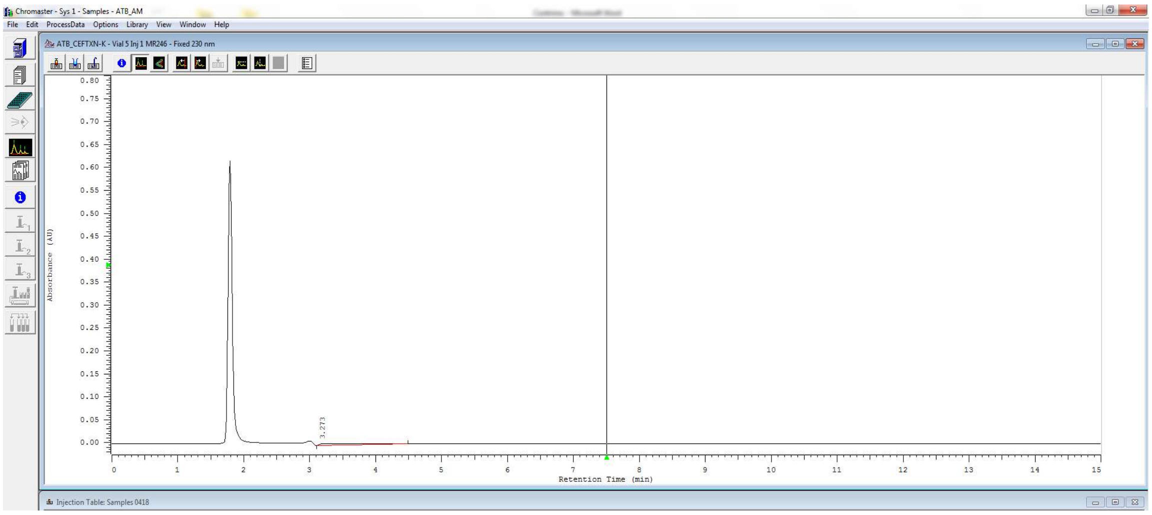

Figure S3F. Sample chromatogram for Ceftriaxone

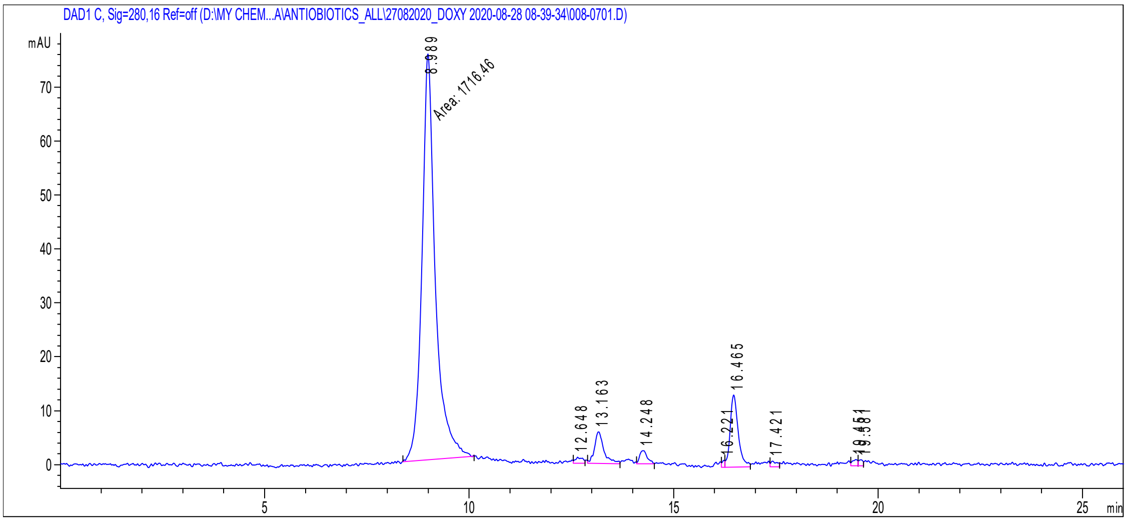

Figure S3G. Sample chromatogram for Doxycycline

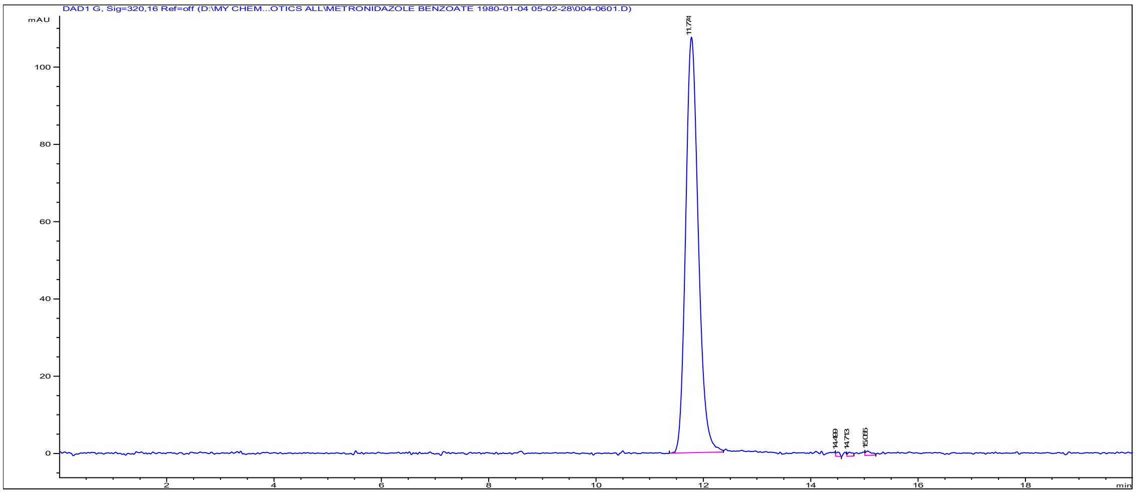

Figure S3H. Sample chromatogram for Metronidazole

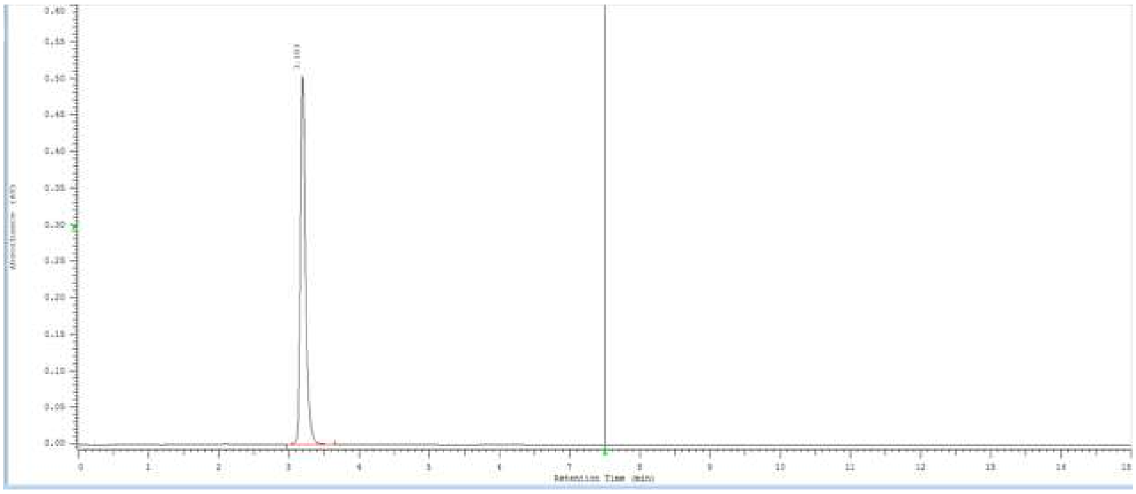

Figure S3I. Sample chromatogram for Ampicillin

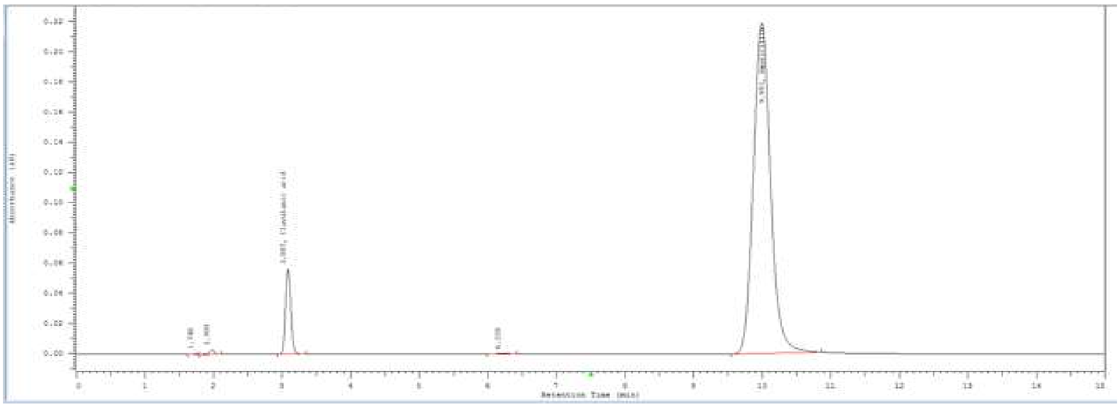

Figure S3J. Sample chromatogram for Amoxicillin + clavulanic acid

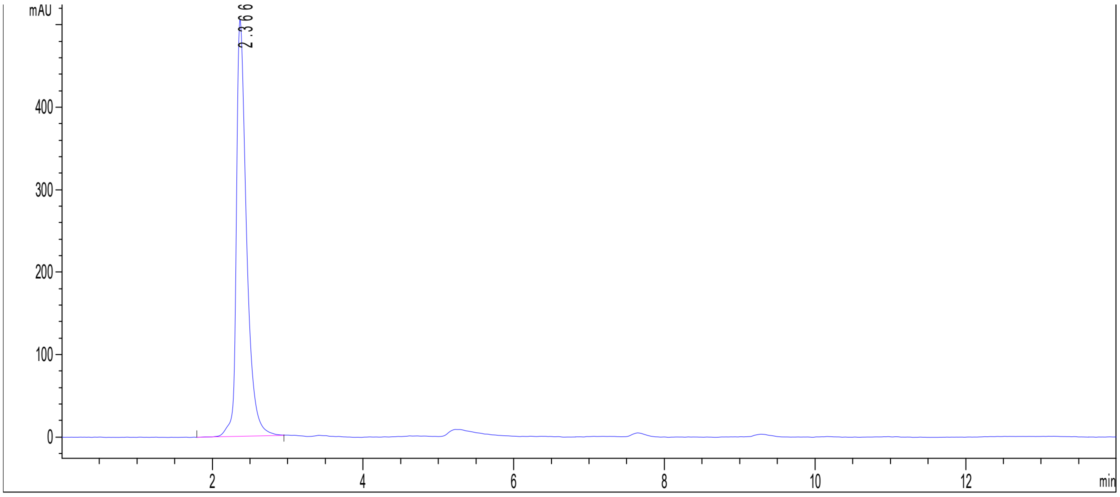

Figure S3K. Sample chromatogram for Erythromycin

Table S1. Summary of analyzed batches and HPLC results.

No	Generic names	Distribution of the 232 batches analyzed and Results of HPLC assay test																Percent of batches that failed HPLC assay test		
		Tablets		Capsules		Powders for oral suspension		oral Suspension		Powder for injection		Solutions		Number of batches analysed				within the total batches of the antibiotic	within the total substandard batches	Within the total number of batches
		Pass	Fail	Pass	Fail	Pass	Fail	Pass	Fail	Pass	Fail	Pass	Fail	Total	%	Pass	Fail			
1	Amoxicillin			31	0	24	7							62	26.7%	55	7	11.3%	36.8%	3.0%
2	Co-trimoxazole	19	4					13	1					37	15.9%	32	5	13.5%	26.3%	2.2%
3	Cloxacillin			19	2	8				2				31	13.4%	29	2	6.5%	10.5%	0.9%
4	Erythromycin	14	0			10	1							25	10.8%	24	1	4.0%	5.3%	0.4%
5	Ciprofloxacin	20	0											20	8.6%	20	0	0.0%	0.0%	0.0%
6	Doxycycline	13	0	6	0									19	8.2%	19	0	0.0%	0.0%	0.0%
7	Metronidazole	5	0					7	2			3	0	17	7.3%	15	2	11.8%	10.5%	0.9%
8	Phenoxymethyl -penicillin	7	0											7	3.0%	7	0	0.0%	0.0%	0.0%
9	Ampicillin									4	1			5	2.2%	4	1	20.0%	5.3%	0.4%
10	Ceftriaxone									3	1			4	1.7%	3	1	25.0%	5.3%	0.4%
11	Cefotaxime									3	0			3	1.3%	3	0	0.0%	0.0%	0.0%
12	Amoxicillin + Clavulanic acid	2	0											2	0.9%	2	0	0.0%	0.0%	0.0%
	Total	80	4	56	2	42	8	20	3	12	2	3	0	232	100%	213	19	8.2%	100.0%	8.2%

No	Generic names	Distribution of the 232 batches analyzed and Results of HPLC assay test																Percent of batches that failed HPLC assay test		
		Tablets		Capsules		Powders for oral suspension		oral Suspensions		Powder for injection		Solutions		Number of batches analysed				within the total batches of the antibiotic	within the total substandard batches	Within the total number of batches
		Pass	Fail	Pass	Fail	Pass	Fail	Pass	Fail	Pass	Fail	Pass	Fail	Total	%	Pass	Fail			
1	Amoxicillin			31	0	24	7							62	26.7%	55	7	11.3%	36.8%	3.0%
2	Co-trimoxazole	19	4					13	1					37	15.9%	32	5	13.5%	26.3%	2.2%
3	Cloxacillin			19	2	8				2				31	13.4%	29	2	6.5%	10.5%	0.9%
4	Erythromycin	14	0			10	1							25	10.8%	24	1	4.0%	5.3%	0.4%
5	Ciprofloxacin	20	0											20	8.6%	20	0	0.0%	0.0%	0.0%
6	Doxycycline	13	0	6	0									19	8.2%	19	0	0.0%	0.0%	0.0%
7	Metronidazole	5	0					7	2			3	0	17	7.3%	15	2	11.8%	10.5%	0.9%
8	Phenoxymethyl-penicillin	7	0											7	3.0%	7	0	0.0%	0.0%	0.0%
9	Ampicillin									4	1			5	2.2%	4	1	20.0%	5.3%	0.4%
10	Ceftriaxone									3	1			4	1.7%	3	1	25.0%	5.3%	0.4%
11	Cefotaxime									3	0			3	1.3%	3	0	0.0%	0.0%	0.0%
12	Amoxicillin + Clavulanic acid	2	0											2	0.9%	2	0	0.0%	0.0%	0.0%
	**Total**	**80**	**4**	**56**	**2**	**42**	**8**	**20**	**3**	**12**	**2**	**3**	**0**	232	100%	213	19	8.2%	100.0%	8.2%

Table S2. Adapted tool for visual inspection.

Sample code: ……………………………………

Site description
PROVINCE: ………………………. DISTRICT: ……………………………
PHARMACY: …………………………………

Sample description
Generic name: …………………………. Brand name: ………………………….
Dosage form: …………………………… Strength: ………………………………
Manufacturer: ………………………….. Country of origin: ……………………
Manufacturing date: …………………… Expiry date: …………………………..
Batch number: ………………………….

A. Visual inspection parameters

PACKAGING
Any medicine should be packaged in a container, which can be anything from a glass bottle to a blister pack, to a tube of glass, plastic or metal. A folding carton bearing the label very often protects the container. Check the type of packaging and compare it to known containers for the same product from the same manufacturer. The packaging and the labelling of pharmaceutical products is a very complex and expensive business. Thus, the process and the quality of packaging material are difficult to counterfeit. This is why a thorough visual inspection could be an important screening step for product quality control. However, producers of counterfeit products are quick to copy special labelling and holograms

1. Container and Closure

	Yes	No	Other observations
Do the container and closure protect the product from the outside environment; e.g., is the container properly sealed?			
Do they assure that the product will meet the proper specifications throughout its shelf life?			
Are the container and the closure appropriate for the product inside?			
Is the container safely sealed?			

2. Label
The information written on the label is very important. The information can be printed on a label adhered to the container, or printed directly onto the container itself, but all information must be legible and indelible.

	Yes	No	Other observations
If there is a carton protecting the container, does the label on the carton match the label on the container?			
Is all information on the label legible and indelible?			
Is the list of excipients provided?			

3. The trade (brand) name

	Yes	No	Other observations
Is the trade name spelled correctly?			

For blister or foil strip-packed products, is the trade name indelibly impressed or imprinted onto the strip?			

4. The active ingredient name (scientific name/generic name) and excipients:

	Yes	No	Other observations
Is the active ingredient name spelled correctly?			
Is the list of excipients provided?			

5. The manufacturer's full address:

All manufacturers are required by international law to print their complete address on the label. Many companies making substandard or counterfeit products do not have a traceable address on the label.

	Yes	No	Other observations
Is the manufacturer's full address legible and correct?			

6. The medicine strength (mg/unit):

	Yes	No	Other observations
Is the strength - the amount of active pharmaceutical ingredient per unit - clearly stated on the label?			
For blister or foil strip-packed products, is the medicine strength indelibly impressed or imprinted onto the strip?			

7. The dosage form (e.g., tablet/capsule):

	Yes	No	Other observations
Is the dosage form clearly indicated on the container label?			
Does the dosage form stated on the label match the actual dosage form of the medication?			

8. The number of units per container:

	Yes	No	Other observations
Does the number of dosage units listed on the label match the number of dosage units stated on the container?			

9. Dosage statement (if appropriate)

	Yes	No	Other observations
Is the dosage clearly indicated on the label?			

Is the dosage stated on the label appropriate for the medicine in this form and strength?			

10. The batch (or lot) number:
Medicines with the same batch/lot number are expected to be equivalent. In a continuous process, a batch corresponds to a defined portion of the production, based on time or quantity. Products from the same batch number should have the same history of manufacturing, processing, packing, and coding. All product quality-control testing should be based on batch/lot numbers.

	Yes	No	Other observations
For blister or foil strip-packed medicines, is the batch number indelibly impressed or imprinted onto the strip?			

11. The date of manufacture and the expiry date:
An expired product should not be sold under any circumstances.

	Yes	No	Other observations
Are the manufacture dates clearly indicated on the label?			
Are the expiry dates clearly indicated on the label?			
For blister or foil strip-packed products, is the expiry date indelibly impressed or imprinted onto the strip?			

12. Storage information:

	Yes	No	Other observations
Are the storage conditions indicated on the label?			
Has the product been properly stored?			

13. Leaflet or package insert:
All product packs contain a leaflet explaining dosage, the medicine content, the adverse effects, the medicine's actions, and how the medicine should be taken. The only exceptions are where the packaging includes all the information that would otherwise be in the leaflet

	Yes	No	Other observations
Is the package insert printed on the same-colored or same-quality paper as the original (If available to compare) or does it look familiar?			
Is the ink on the package insert or packaging smudge-proof?			
Does the information on the package insert match the information on the product container?			

Is the list of excipients provided?

14 Physical characteristics

14.1. Physical characteristics of tablets/capsules

All types of medicines can be and have been counterfeited, from cough syrups to injections. As mentioned above, it is important to check the packaging of these medicines. Additionally, tablets or capsules can be checked for signs of moisture, dirty marks, abrasion, erosion, cracks, or any other adulteration.

	Yes	No	Other observations
14.1.1 Uniformity of Shape:<br>Are the tablets/capsules uniform in shape?			
14.1.2 Uniformity of Size:<br>Are the tablets/capsules uniform in size?			
14.1.3 Uniformity of Color:<br>Are the tablets/capsules uniform in color?			
14.1.4 Uniformity of Texture:<br>Tablets can be film-coated, sugar-coated or enteric-coated.<br>Do the tablets have a uniform coating?			
Is the base of the tablets fully covered?			
Are the tablets uniformly polished, free of powder, and non-sticking?			
14.1.5 Markings (scoring, letters, etc):<br>Are markings uniform and identical?			
14.1.6 Breaks, Cracks and Splits:<br>Are the tablets/capsules free of breaks, cracks, splits or pinholes?			
14.1.7 Embedded surface spots or contamination:<br>Are the tablets/capsules free of embedded<br>surface spots and foreign particle contamination?			
14.1.8 Presence of empty capsules in the case of a sample of capsules:<br>Is the sample examined free of empty capsules?			
14.1.9 Smell<br>Does the medicine smell peculiar?			

14.2. Physical characteristics of powders (for suspension or for injection)

	Yes	No	Other observations
14.2.1 Uniformity of texture:<br>is the powder free of crystals and blocs?			
14.2.2 Presence of foreign particles:<br>Is the powder free of foreign particles?			

14.3. Physical characteristics of Liquids

14.3.1. Physical characteristics of Solutions for injection

	Yes	No	Other observations
Is the color normal?			
Are foreign particles absent?			
Are precipitates absent?			
Is leakage absent?			

14.3.2. Physical characteristics of Solution for syrups

	Yes	No	Other observations
Is the color normal?			
Does the syrup look viscous?			
Are precipitates absent?			
Is leakage absent?			

14.3.3. Physical characteristics of Suspensions

	Yes	No	Other observations
Is the color normal?			
Is caking absent?			
Is leakage absent?			

B. Reporting Counterfeit Medicines

If, after carrying out the above visual inspection, you suspect you have discovered a counterfeit medicine, you should report this immediately to your local health authority.

Table S3. HPLC chromatographic conditions and sample preparation.

No	Molecule	Chromatographic Conditions
1	Doxycycline	HPLC method from the United States Pharmacopoeia (USP) was carried out using an HPLC-UV Agilent 1200 series equipped with a degasser G1322A, pump G1311A, autosampler G1329A, thermostat G1316A, and detector G1315. An L7 LiChrosorb® RP-8 column (250x4 mm, 5µm) was used for separation. Samples were eluted with KH_2PO_4 0.01M, pH = 8.5 buffer (Mobile phase A) and Methanol (Mobile phase B) under the following gradient conditions: 0 min, 10%B; 0-2 min (10%B); 2-4 min (40%B); 4-6 min (10%B); 6-9 min (10%B). The injection volume was 5 µL, the column was kept at 60 °C, the flow rate was 0.6 mL/min, and the detection wavelength was 280 nm.
2	Erythromycin	HPLC analysis was carried out with reference to USP, using an HPLC-UV Agilent 1200 series equipped with degasser G1322A, pump G1311A, autosampler G1329A, thermostat G1316A, and detector G1315D. Samples were eluted through a 250 x 4.6 mm column, in a gradient mode with the following gradient profile and mobile phase compositions: 0-10 min (90%A/10%B), 10-18 min (0%A/100%B). The mobile phase A was 10% CH_3CN and the mobile phase B was 75% CH_3CN. The mobile phase flow rate was set at 1.3 mL/min and the sample injection volume was 50 µL. The column was conditioned at 50°C. USP reference standards were used.
3	Cloxacillin	With reference to USP, HPLC analysis was carried out using an HPLC-UV Agilent 1200 series equipped with a degasser G1322A, pump G1311A, autosampler G1329A, thermostat G1316A, and detector G1315D. An L1 Luna Omega Polar C_{18} (250x4.6 mm, 5µm) column was used for the separation. Samples were eluted isocratically with Acetonitrile / KH2PO40.01M Buffer pH = 6.8 (20/80). The injection volume was 20µL. The column temperature was 25°C, the flow rate was 1 mL/min, and the detection wavelength was 225 nm.
4	Phenoxymethyl- penicillin	Reference made to USP, HPLC analysis was carried out using an HPLC-UV Agilent 1200 series equipped with a degasser G1322A, pump G1311A, autosampler G1329A, thermostat G1316A, and detector G1315D. An L1 Luna Omega Polar C_{18} (250x4.6 mm, 5µm) column was used for the separation. Samples were eluted isocratically with Acetonitrile, glacial acetic acid, and water (350: 5.75: 650). The injection volume was 10 µL, the temperature was 25°C, the flow rate was 1 mL/min, and the detection wavelength was 254nm.

No	Molecule	Chromatographic Conditions
5	Cotrimoxazole	Using USP as the reference, HPLC analysis was carried out using an HPLC-UV Agilent 1200 series equipped with a degasser G1322A, pump G1311A, autosampler G1329A, thermostat G1316A, and detector G1315D. An L1 Luna Omega Polar C_{18} (250x4.6 mm, 5µm) column was used for the separation. Samples were eluted isocratically with Acetonitrile/H2O/Triethylamine in the ratio of 20/79.9/0.1 respectively with a pH of 5.9. The injection volume was 20 µL, the analysis was performed at 25°C, the flow rate was 2ml/min, and the detection wavelength was 254nm.
6	Ciprofloxacin	Referring to the USP, HPLC analysis was carried out using an HPLC-UV Agilent 1200 series equipped with Degasser G1322A, pump G1311A, autosampler G1329A, thermostat G1316A, and detector G1315D. An L1 Luna Omega Polar C_{18} (250x4.6 mm, 5µm) column was used for the separation. Samples were eluted isocratically with Mobile phase: Acetonitrile: phosphoric acid 0.025 M pH 3 (13:87). The injection volume was 10 µL, the analysis temperature was 30°C, the flow rate was 1.5ml/min, and the detection wavelength was 278nm.
7	Cefotaxime	HPLC analysis was carried out with reference to the USP, using an HPLC-UV Agilent 1200 series equipped with degasser G1322A, pump G1311A, autosampler G1329A, thermostat G1316A, and detector G1315D. An L1 Luna Omega Polar C_{18} (250x4.6 mm, 5µm) column was used for the separation. Samples were eluted in a gradient mode with two mobile phases A and B as follows: 0-7 min (0%B); 7-16 min (20%B); 16-51 min (100%B); 51-56 min (0%B). Mobile phase A: Methanol and buffer (14:86). Mobile phase B: Methanol and buffer (40:60). Buffer: 7.1 g/L of anhydrous dibasic sodium phosphate in water, adjusted with phosphoric acid to a pH of 6.25. The injection volume was 10µL, the temperature was 30°C, the flow rate was 1 mL/min, and the detection wavelength was 235nm.
8	Amoxicillin	USP served as the reference to carry out the HPLC analysis, using an HPLC-UV HITACHI and USP amoxicillin RS was prepared in buffer. An L1 Luna Omega Polar C_{18} (250x4.6 mm, 5µm) column was used for the separation. Samples were eluted in isocratic mode with a mobile phase composed by acetonitrile and buffer KH_2PO_4 0.01M, pH=5 (1:24). The buffer was prepared by dissolving 6.8 g/L of monobasic potassium phosphate in water, adjusting with 45% potassium hydroxide TS to a pH<br>of 5.0. The injection volume was 10 µL, the temperature was 25 °C, the flow rate was 1.5ml/min, and the detection wavelength was 230 nm.

No	Molecule	Chromatographic Conditions
9	Metronidazole	Reference made to USP, HPLC analysis was carried out using an HPLC-UV Agilent 1200 series equipped with a degasser G1322A, pump G1311A, autosampler G1329A, thermostat G1316A, and detector G1315D. An L7 LiChrosorb® RP-8 column (250x4 mm, 5µm) was used for separation. Samples were eluted in isocratic mode with Methanol and water (20:80). The injection volume was 10µL, the temperature was 30 °C, the flow rate was 1ml/min, and the detection wavelength was 254 nm. Standard solution: 0.5 mg/mL of USP Metronidazole RS in the mobile phase.
10	Ampicillin	With reference to the USP, HPLC analysis was carried out using an HPLC-UV HITACHI. An L1 Luna Omega Polar C_{18} (250x4.6 mm, 5µm) column was used for the separation. Samples were eluted isocratically with a mobile phase composed by water, Acetonitrile, 1M monobasic potassium phosphate buffer and 1N glacial acetic acid in the ratio of 90.9:8:1:0.1 respectively. The injection volume was 20 µL, the temperature was 40°C, the flow rate was 2ml/min, and the detection wavelength was 254 nm.
11	Ceftriaxone	Reference was made to the USP to perform HPLC analysis using an HPLC-UV HITACHI. An L1 Luna Omega Polar C_{18} (250x4.6 mm, 5µm) column was used for the separation. Samples were eluted isocratically with a mobile phase prepared by dissolving 2.0 g each of tetradecylammonium bromide and tetraheptylammonium bromide in a mixture of 440 mL of water, 55 mL of Buffer, 5.0 mL of Solution C and 500 mL of acetonitrile, the injection volume was 20 µL, the temperature was 25°C, the flow rate was 1.5ml/min, and the detection wavelength was 254 nm. A 0.3mg/mL USP ceftriaxone RS was used as the standard solution.
12	Amoxicillin + clavulanic acid	USP served as the reference to carry out the HPLC analysis, using an HPLC-UV HITACHI. An L1 Luna Omega Polar C_{18} (250x4.6 mm, 5µm) column was used for the separation. Samples were eluted in isocratic mode with Methanol and Buffer (1:19). The buffer was prepared by dissolving 7.8 g of monobasic sodium phosphate in 900 mL of water and adjusting the pH value of 4.5 with phosphoric acid, then toping up to 1000 mL with water. The injection volume was 20 µL, the temperature was 25 °C, the flow rate was 2ml/min, and the detection wavelength was 220 nm.
